# *Saccharina japonica*-Derived Fucoidan Protects Intestinal Immune Homeostasis by Modulating Dendritic Cell Function and Alleviating LPS-Induced Acute Enteritis

**DOI:** 10.3390/md24080274

**Published:** 2026-08-06

**Authors:** Peiru Li, Hongyuan Ma, Muyan Li, Zilan Liu, Xuanru Zhou, Jian Li, Yijian Wu, Ping Liu

**Affiliations:** 1 Fujian Key Laboratory of Traditional Chinese Veterinary Medicine and Animal Health, College of Animal Science, Fujian Agriculture and Forestry University, Fuzhou 350002, China; 2 School of Future Technology, Fujian Agriculture and Forestry University, Fuzhou 350002, China; 3 University Key Laboratory for Integrated Chinese Traditional and Western Veterinary Medicine and Animal Healthcare, College of Animal Science, Fujian Agriculture and Forestry University, Fuzhou 350002, China

**Keywords:** fucoidan, dendritic cells, Th1/Th17/Treg balance, acute enteritis, intestinal barrier

## Abstract

Fucoidan (FUC) exhibits immunomodulatory activity; however, its effects on intestinal mucosal immunity through dendritic cell (DC)-mediated regulation remain unclear. In this study, fucoidan was extracted from *Saccharina japonica* by hot-water extraction and characterized by chemical composition analysis, gel permeation chromatography (GPC), and Fourier-transform infrared spectroscopy (FT-IR). Bone marrow-derived DCs were used to evaluate the effects of FUC on DC maturation and immune function. An LPS-induced acute enteritis mouse model was used to assess intestinal injury, barrier function, and DC-mediated T/B cell immune responses. Structural analysis confirmed that purified FUC has the sulfated polysaccharide characteristics. FUC promoted DC maturation and enhanced antigen-presenting capacity. In LPS-induced enteritis, FUC reduced IL-1β, IL-6, and TNF-α levels and improved intestinal barrier integrity by restoring the mRNA expression of tight-junction-related genes. Mechanistically, FUC regulated the excessive activation of the TLR4/MyD88/NF-κB pathway, increased TGF-β and IFN-γ expression, modulated Th1/Th17/Treg immune balance, and promoted B cell homing and sIgA secretion. FUC regulates DC-mediated immune responses, repairs intestinal mucosal barrier function, and restores the intestinal immune microenvironment, thereby alleviating LPS-induced intestinal inflammation and maintaining intestinal homeostasis.

## 1. Introduction

Plant-derived polysaccharides exhibit various biological activities, including immunomodulatory, anti-inflammatory, antibacterial, and antiviral effects [1]. They can regulate intestinal mucosal immune responses and help maintain intestinal microbial homeostasis [2]. With increasing interest in marine resources, bioactive polysaccharides derived from marine organisms have received growing attention. Fucoidan (FUC) is a sulfated polysaccharide with various biological activities, including antioxidant, antitumor, lipid metabolism-regulating, and immunomodulatory effects [3]. These activities are closely associated with its unique chemical structure.

FUC has been reported to regulate immune responses through multiple mechanisms. It enhances macrophage phagocytosis and antigen presentation by increasing the expression of surface molecules such as CD80 and CD86 [4]. In addition, FUC improves NK cell cytotoxicity and promotes the secretion of perforin and granzyme B, thereby enhancing antitumor and antiviral immune responses [5]. FUC also promotes CD4^+^ T cell differentiation toward the Th1 phenotype by increasing IFN-γ and IL-12 production, while reducing Th2-related cytokine expression [6]. These findings indicate that FUC can regulate immune function by acting on different immune cell populations, including macrophages, NK cells, and T cells. Moreover, FUC has been shown to alleviate inflammation and tissue injury in acute colitis models [7].

Dendritic cells (DCs) are key antigen-presenting cells in intestinal mucosal immunity and play an important role in determining immune responses [8]. DCs regulate T cell differentiation, including Th1, Th17, and regulatory T cell (Treg) responses, through cytokine secretion and the expression of costimulatory molecules [9]. They also contribute to B cell activation and antibody production [10]. Therefore, the regulation of DC function is an important aspect in understanding how immunomodulatory agents affect intestinal mucosal immunity.

The intestinal mucosal immune system is the largest mucosa-associated lymphoid tissue in the body. It consists of intraepithelial lymphocytes, Peyer’s patches, mesenteric lymph nodes, lamina propria immune cells, and various immune mediators [11,12]. These components work together to maintain intestinal immune homeostasis. Diffuse immune cells in the intestinal epithelium and lamina propria directly participate in immune defense by migrating to antigen-exposed sites and mediating immune responses. Organized lymphoid structures, such as Peyer’s patches and mesenteric lymph nodes, serve as important sites for antigen recognition and immune activation. DCs, macrophages, and microfold cells in these structures capture and present antigens, thereby initiating adaptive immune responses [13].

As important regulators of intestinal immunity, DCs are essential for maintaining intestinal immune homeostasis. These professional antigen-presenting cells are mainly located in Peyer’s patches and the intestinal lamina propria [14]. They continuously monitor intestinal environmental changes through pattern recognition receptors (PRRs) and serve as a link between innate and adaptive immunity [15]. Immature DCs have strong antigen uptake ability. After recognizing pathogens, they undergo maturation, characterized by increased CCR7 expression, migration to lymphoid tissues, and enhanced expression of MHC molecules and costimulatory molecules such as CD80/CD86. These changes allow DCs to effectively activate CD4^+^ and CD8^+^ T cells and initiate antigen-specific immune responses [16,17]. In the intestinal mucosa, DCs are widely distributed in the lamina propria and associated lymphoid tissues, where they regulate both immune homeostasis and inflammatory responses [18].

However, whether FUC regulates intestinal mucosal immunity through direct modulation of DC function remains unclear. The underlying molecular mechanisms also require further investigation. In this study, in vitro DC experiments and an LPS-induced mouse enteritis model were used to evaluate the effects of FUC on DC maturation and DC-mediated T and B cell immune responses. This study provides new insights into the immunoregulatory mechanisms of marine-derived polysaccharides and supports the potential application of FUC as a natural immunomodulatory agent.

## 2. Results

### 2.1. Physicochemical Properties and Structural Characterization of FUC

**Figure 1:**
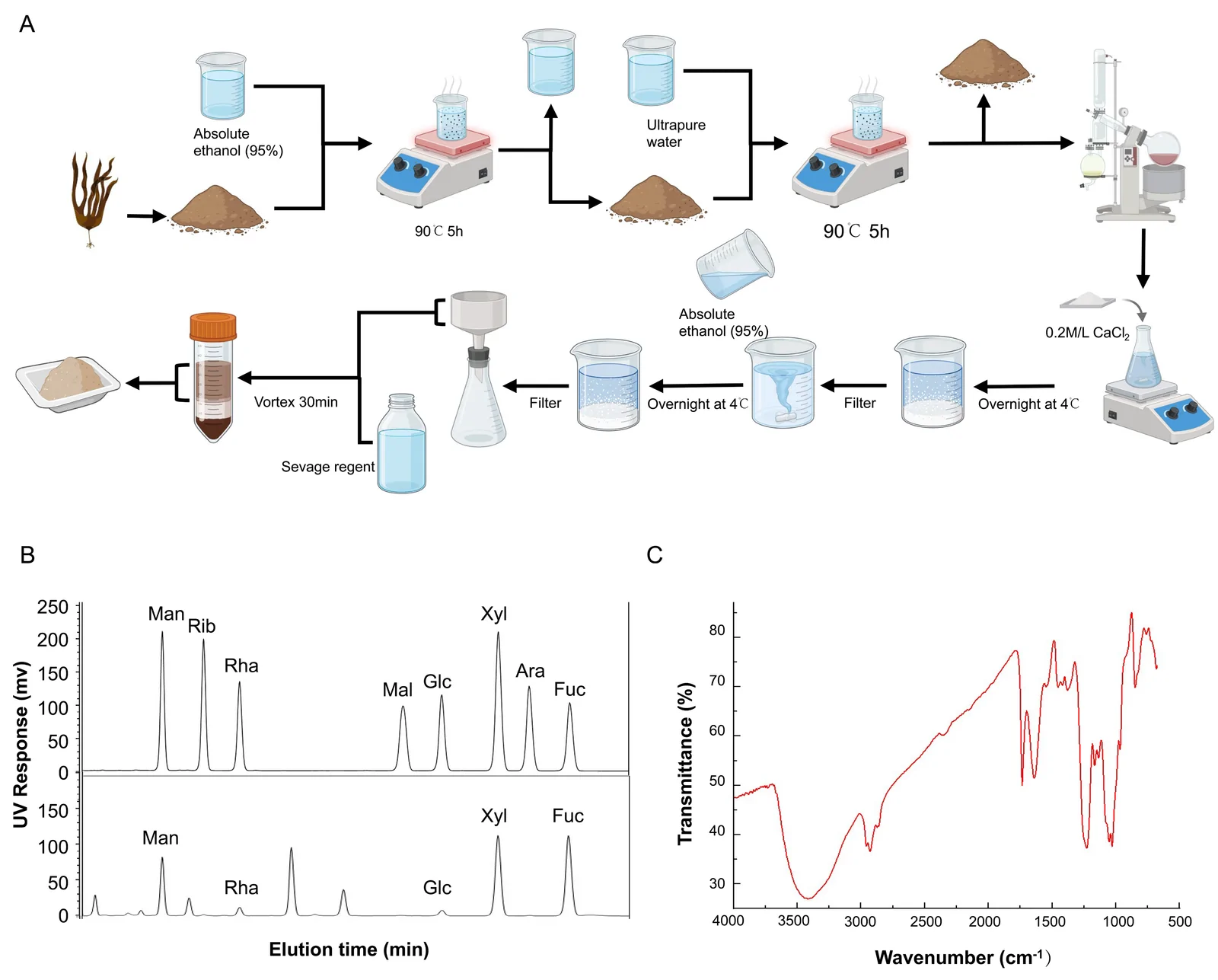
Physicochemical Properties and Structural Characterization of FUC. (**A**) Flow chart of crude fucoidan extraction process. (**B**) Monosaccharide composition of FUC determined by HPLC. (**C**) FT−IR spectrum of FUC.

FUC was successfully extracted from kelp using a hot-water extraction method, and the extraction procedure is illustrated in Figure 1A. The FUC isolated from *Saccharina japonica* contained total carbohydrates 79.58%, fucose 19.58%, uronic acid 6.51%, and sulfate groups 24.16%. Its average molecular weight was 73.5 kDa. Monosaccharide analysis showed that FUC was mainly composed of mannose (Man), rhamnose (Rha), glucose (Glc), galactose (Gal), xylose (Xyl), and fucose (Fuc), with a molar ratio of 5.60:1.35:1.00:11.16:2.34:15.86. Fucose was the predominant monosaccharide component (Figure 1B).

The FT-IR spectrum of FUC showed characteristic absorption bands of polysaccharides (Figure 1C). The broad band at 3406 cm^−1^ was assigned to O–H stretching vibration. The peak at 2924 cm^−1^ was attributed to C–H stretching vibration and was likely associated with the CH_3_ groups of fucose residues. The absorption bands at 1736 and 1639 cm^−1^ were assigned to asymmetric stretching vibrations of non-esterified carbonyl groups (C=O), indicating the presence of uronic acids. In addition, the absorption band at 1227 cm^−1^ was attributed to asymmetric S=O stretching vibration, while the band at 852 cm^−1^ was assigned to C–O–S stretching vibration. These signals confirmed the presence of sulfate groups. Collectively, the FT−IR results indicated that FUC is an anionic sulfated polysaccharide.

### 2.2. FUC Promotes BMDC Maturation and Modulates Immune Function in Association with TLR4/MyD88/NF-κB Signaling

**Figure 2:**
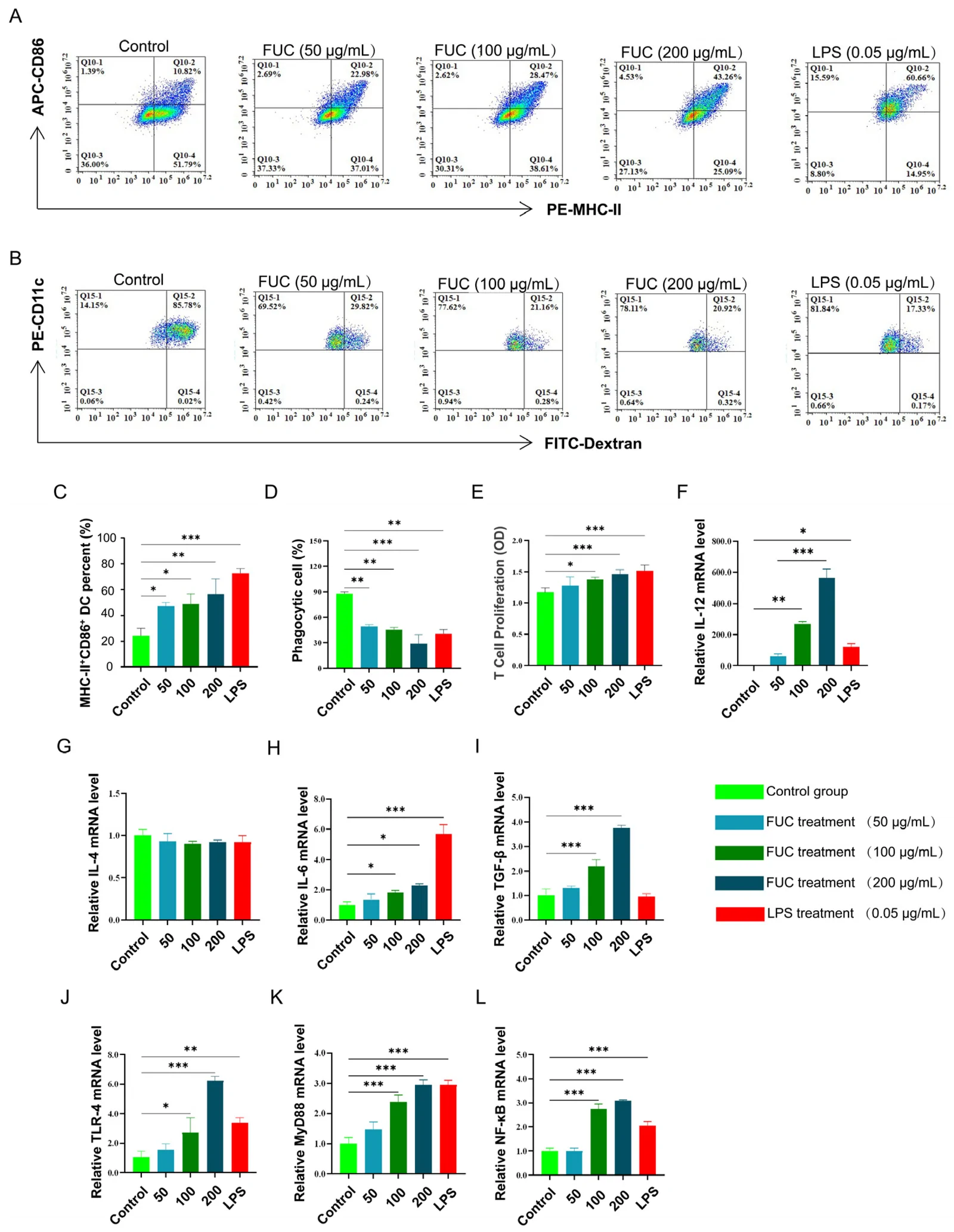
FUC induces maturation and immunomodulatory function of BMDCs through the TLR4/NF-κB pathway in vitro. (**A**) Flow cytometric analysis of the proportion of MHC-II^+^CD86^+^ DCs after FUC treatment. (**B**) Flow cytometric assessment of DC phagocytic activity after FUC treatment. (**C**) Changes in the proportion of MHC-II^+^CD86^+^ DCs following FUC treatment. (**D**) Effects of FUC on DC phagocytic function. (**E**) Effects of FUC-stimulated DCs on T lymphocyte proliferation. (**F**–**I**) Cytokine expression levels in DCs. (**J**–**L**) Gene expression of DC-related signaling pathways. * *p* < 0.05, ** *p* < 0.01, *** *p* < 0.001. Density plots were generated using FlowJo software v10, where color intensity represents the relative density of events.

MHC-II and CD86 double-positive cells were used as markers of DC maturation. Compared with the control group, treatment with 50, 100, and 200 μg/mL FUC significantly increased the proportion of MHC-II^+^CD86^+^DCs in a concentration-dependent manner (*p* < 0.05; Figure 2A,C). These results indicate that FUC promoted the maturation of mouse BMDCs and enhanced their antigen-presenting capacity.

Immature DCs have strong phagocytic activity. Compared with the control group, FUC treatment significantly reduced FITC-dextran uptake by DCs by 55.96%, 64.62%, and 64.86% at concentrations of 50, 100, and 200 μg/mL, respectively (*p* < 0.05; Figure 2B,D). This reduction was consistent with the transition of DCs from an immature to a mature phenotype.

T cell proliferation was used to assess the antigen-presenting function of DCs. DCs were co-cultured with mouse splenic T cells at a ratio of 1:5 for 72 h. Compared with the control group, DCs treated with 100 or 200 μg/mL FUC significantly promoted T cell proliferation (*p* < 0.05; Figure 2E). These data further support the ability of FUC to enhance DC-mediated T cell activation.

Compared with the control group, treatment with 100 or 200 μg/mL FUC for 48 h significantly increased the mRNA expression of IL-12, IL-6, and TGF-β in DCs (*p* < 0.05; Figure 2F–I). No significant change was observed in IL-4 expression. These results suggest that FUC may modify the DC-derived cytokine environment associated with Th1- and Th17-related immune responses.

To explore the molecular basis of FUC-induced DC activation, the expression of TLR signaling-related molecules was examined. Compared with the control group, 100 and 200 μg/mL FUC significantly increased TLR4 mRNA expression in DCs (*p* < 0.05) and markedly increased the expression of the downstream adaptor protein MyD88 and transcription factor NF-κB (*p* < 0.001; Figure 2J–L). These findings suggest that the TLR4/MyD88/NF-κB pathway may be involved in FUC-induced BMDC maturation and immune regulation.

### 2.3. FUC Alleviates LPS-Induced Acute Enteritis and Improves Intestinal Barrier Function

**Figure 3:**
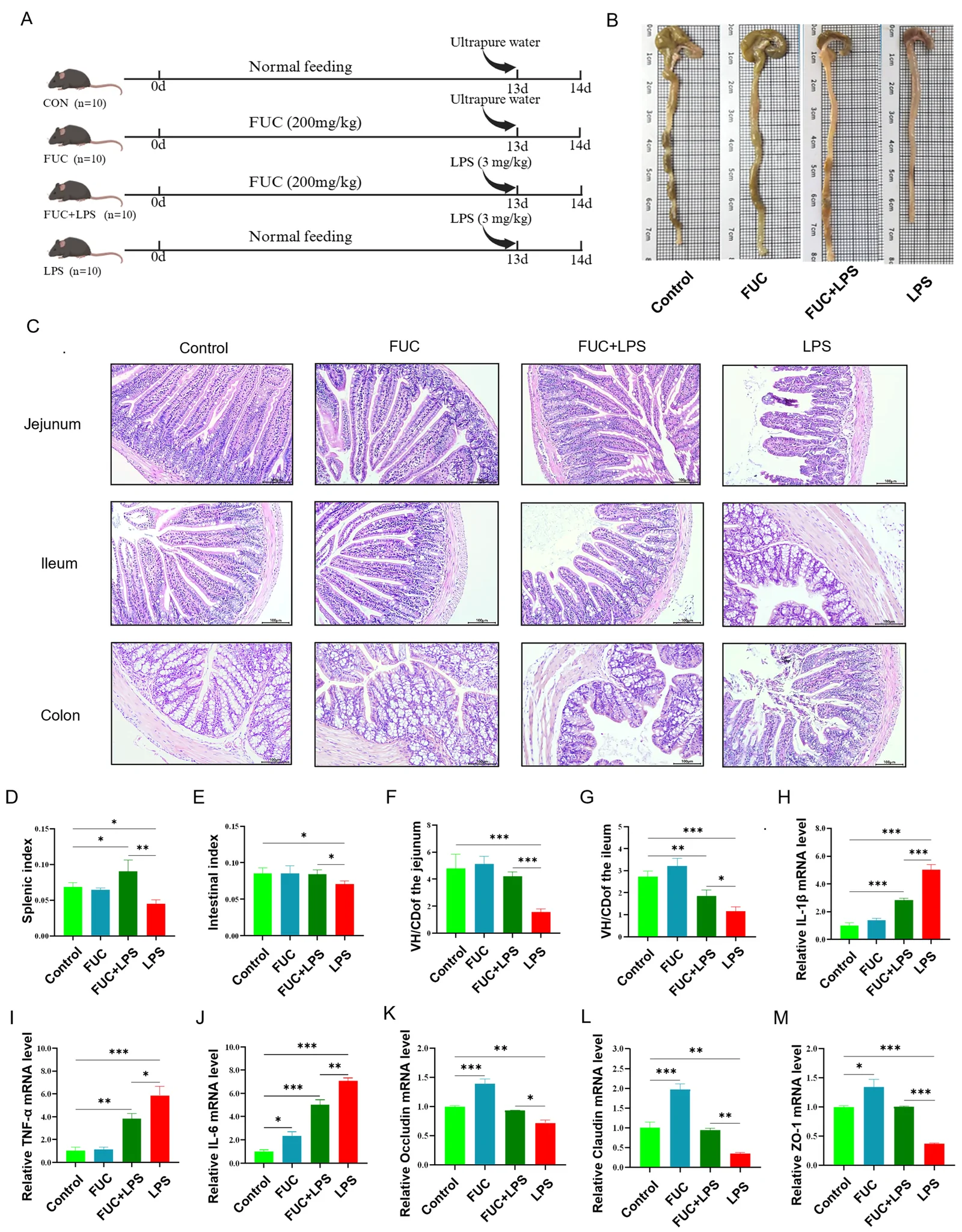
FUC alleviates LPS-induced acute enteritis in mice and improves intestinal barrier function. (**A**) Experimental design of the animal study. (**B**) Intestinal lesions in mice. (**C**) Histological observation of mouse intestinal tissues (H&E staining). (**D**) Spleen-to-body weight ratio in mice. (**E**) Intestine-to-body weight ratio in mice. (**F**) Villus-to-crypt ratio in the jejunum of mice. (**G**) Villus-to-crypt ratio in the ileum of mice. (**H**–**J**) Levels of intestinal inflammatory cytokines in mice. (**K**–**M**) Intestinal epithelial barrier markers in mice. * *p* < 0.05, ** *p* < 0.01, *** *p* < 0.001.

An LPS-induced mouse model of acute enteritis was established to evaluate the effects of FUC on the intestinal mucosal barrier. FUC pretreatment alleviated rectal bleeding, loose stools, and colon shortening induced by LPS (Figure 3B). It also reduced injury to immune organs (Figure 3D,E).

Histological analysis showed that the intestinal structure was intact and villi were well arranged in the control and FUC groups (Figure 3C). In contrast, the LPS group showed typical intestinal injury, including shortened and broken villi, disrupted crypt architecture, and reduced goblet cell numbers. These pathological changes were markedly improved by FUC pretreatment. Morphometric analysis further showed that FUC significantly increased villus height in the jejunum and ileum and reduced crypt depth in the jejunum. As a result, the villus height-to-crypt depth ratio was significantly increased in both the jejunum and ileum (*p* < 0.05; Figure 3F,G).

FUC pretreatment significantly suppressed the LPS-induced increase in intestinal IL-1β, TNF-α, and IL-6 expression (*p* < 0.05; Figure 3H–J). Notably, FUC treatment alone increased IL-6 expression (*p* < 0.05), suggesting that IL-6 may contribute to FUC-mediated regulation of intestinal barrier function under physiological conditions.

LPS-induced acute enteritis disrupts intestinal barrier integrity and increases mucosal permeability. This process may promote bacterial translocation and systemic inflammation. Therefore, qRT-PCR was used to measure the mRNA expression of tight junction-related genes. Compared with the control group, FUC treatment significantly increased the mRNA expression of Occludin, Claudin-1, and ZO-1 in intestinal tissues (*p* < 0.05; Figure 3K–M). LPS significantly dysregulated the expression of these tight junction-related genes. Compared with the LPS group, FUC pretreatment significantly restored the expression of Occludin, Claudin-1, and ZO-1 (*p* < 0.05). These findings indicate that FUC may reinforce the intestinal physical barrier by regulating tight junction protein expression, thereby helping to limit LPS-induced intestinal injury.

### 2.4. FUC Promotes Intestinal DC Maturation and Modulates TLR4-Related Signaling

**Figure 4:**
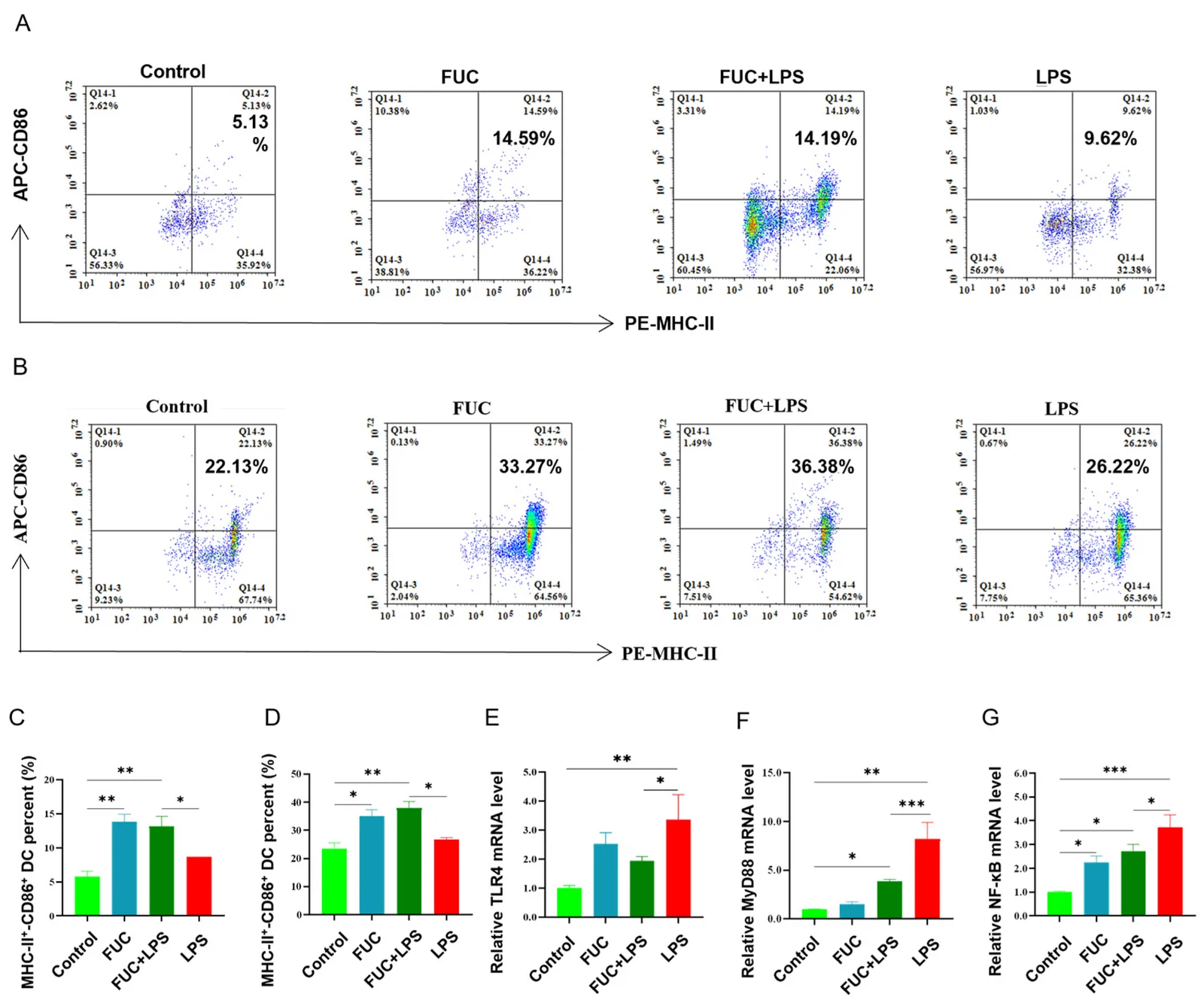
FUC promotes the maturation of intestinal DCs and modulates TLR4-related gene expression. (**A**) Flow cytometric analysis of surface markers on DCs from the intestinal lamina propria. (**B**) Flow cytometric analysis of surface markers on DCs from Peyer’s patches. (**C**) Proportion of DCs in the intestinal lamina propria of mice. (**D**) Number of DCs in Peyer’s patches of mice. (**E**–**G**) Expression levels of genes involved in DC-related signaling pathways. * *p* < 0.05, ** *p* < 0.01, *** *p* < 0.001. Density plots were generated using FlowJo software, where color intensity represents the relative density of events.

To examine the effects of FUC on local intestinal mucosal immunity, DC maturation was evaluated in the intestinal lamina propria and Peyer’s patches. Flow cytometry showed that the proportion of MHC-II^+^CD86^+^ DCs was significantly increased in both the FUC and FUC + LPS groups compared with the control group (*p* < 0.05; Figure 4A–D). In addition, the proportion of mature DCs in the FUC + LPS group was significantly higher than that in the LPS group (*p* < 0.05). These results indicate that FUC promotes intestinal DC maturation under both physiological and inflammatory conditions.

The expression of TLR4 signaling-related genes was further measured in intestinal tissues. Compared with the control group, FUC treatment alone significantly increased TLR4 and NF-κB expression (*p* < 0.05), while MyD88 expression showed an increasing trend without statistical significance (*p* > 0.05; Figure 4E–G). Compared with the LPS group, the expression levels of TLR4, MyD88, and NF-κB were significantly decreased in the FUC+LPS group (*p* < 0.05).

Together, these results indicate that FUC promotes intestinal DC maturation and modulates TLR4-associated signaling in a context-dependent manner. Under physiological conditions, FUC enhanced TLR4- and NF-κB-related signaling. Under LPS challenge, FUC significantly reduced the expression of TLR4, MyD88, and NF-κB relative to the LPS group.

### 2.5. FUC Regulates T Cell-Mediated Cellular Immune Responses

**Figure 5:**
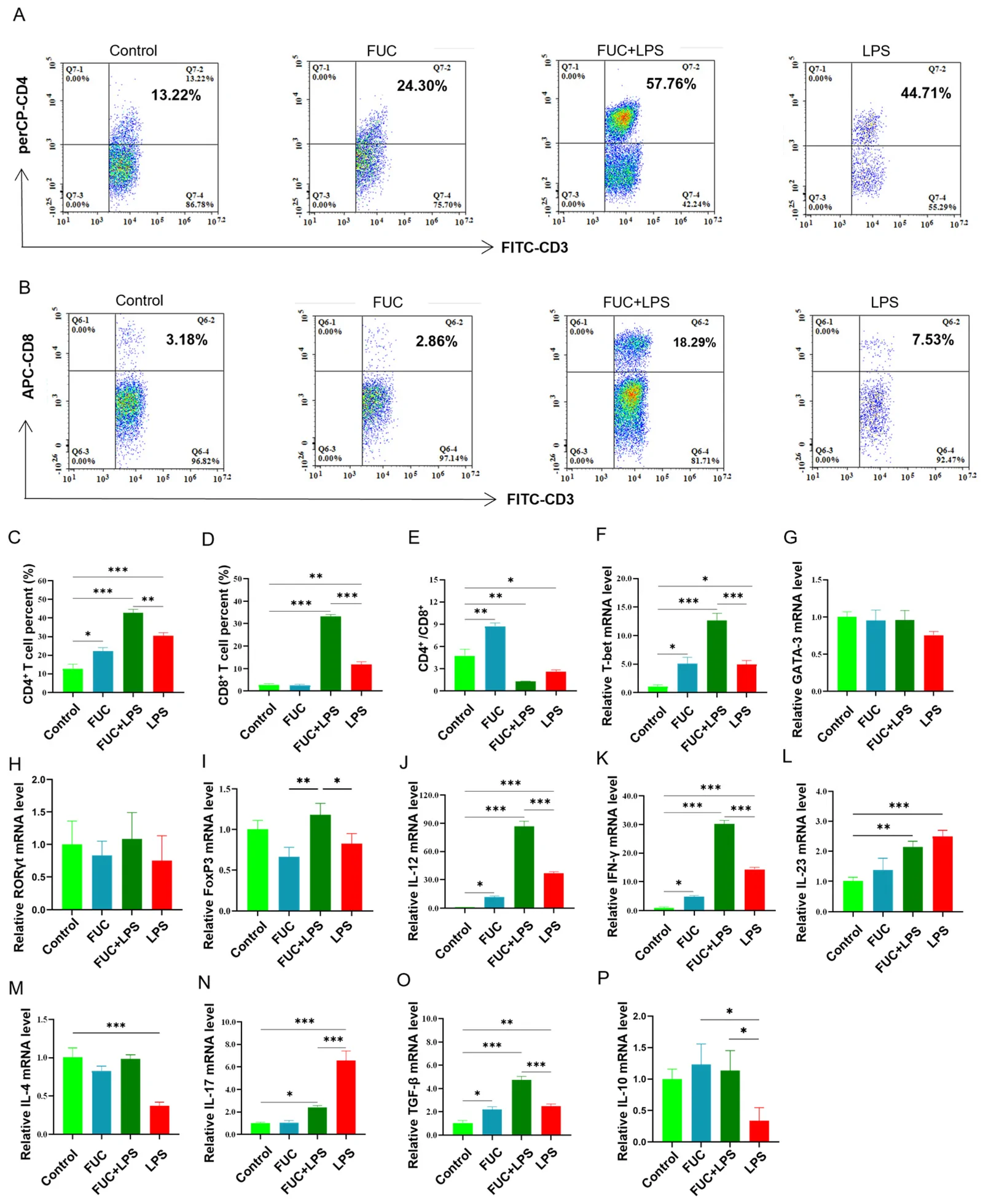
FUC regulates T lymphocyte−mediated cellular immunity. (**A**) Flow cytometric analysis of CD4^+^ T cells in the intestinal tissue of mice. (**B**) Flow cytometric analysis of CD8^+^ T cells in the intestinal tissue of mice. (**C**) Effects of FUC on CD4^+^ T lymphocytes in the small intestine of mice. (**D**) Effects of FUC on CD8^+^ T lymphocytes in the small intestine of mice. (**E**) Effects of FUC on the CD4^+^/CD8^+^ T lymphocyte ratio in the small intestine of mice. (**F**–**I**) Effects of FUC on the expression of key transcription factors in Th cell subsets in mouse intestines. (**J**–**P**) Expression levels of cytokine genes in mouse intestinal tissues. * *p* < 0.05, ** *p* < 0.01, *** *p* < 0.001. Density plots were generated using FlowJo software, where color intensity represents the relative density of events.

Compared with the control group, the proportion of intestinal CD4^+^ T cells was increased by 11.08%, 31.49%, and 44.54% in the FUC, LPS, and FUC + LPS groups, respectively (Figure 5A,C). The proportion of CD4^+^ T cells in the FUC + LPS group was significantly higher than that in the LPS group (*p* < 0.01). In addition, LPS significantly increased the proportion of CD8^+^ T cells (*p* < 0.01), and FUC intervention further increased the proportion of CD8^+^ T cells in the FUC + LPS group (*p* < 0.001; Figure 5B,D).

The expression of key T cell-related transcription factors was then examined (Figure 5F–I). T-bet expression, a Th1-related transcription factor, was significantly higher in the FUC, LPS, and FUC + LPS groups than in the control group (*p* < 0.05). FoxP3 expression, a Treg-associated transcription factor, was significantly higher in the FUC + LPS group than in the FUC and LPS groups (*p* < 0.05). No significant differences were observed in the expression of GATA-3 or RORγt among the groups. These findings suggest that FUC may favor a Th1/Treg-associated immune profile under inflammatory conditions.

The expression of T cell-related cytokines was further analyzed (Figure 5J–P). FUC treatment alone significantly increased IL-12, IFN-γ, and TGF-β expression (*p* < 0.05). LPS treatment significantly increased IL-12, IFN-γ, IL-23, and TGF-β expression (*p* < 0.001), while IL-4 expression was significantly reduced (*p* < 0.001). Importantly, compared with the LPS group, FUC pretreatment significantly increased IL-10, IL-12, IFN-γ, and TGF-β expression (*p* < 0.001) and markedly reduced IL-17 expression. These cytokine changes suggest that FUC may regulate DC-mediated T cell responses by enhancing Th1- and Treg-associated immune regulation while restraining Th17-associated inflammatory activity. This effect may contribute to the improvement of intestinal mucosal inflammation.

### 2.6. FUC Enhances Mucosal Humoral Immunity by Promoting B Cell Accumulation and sIgA Secretion

**Figure 6:**
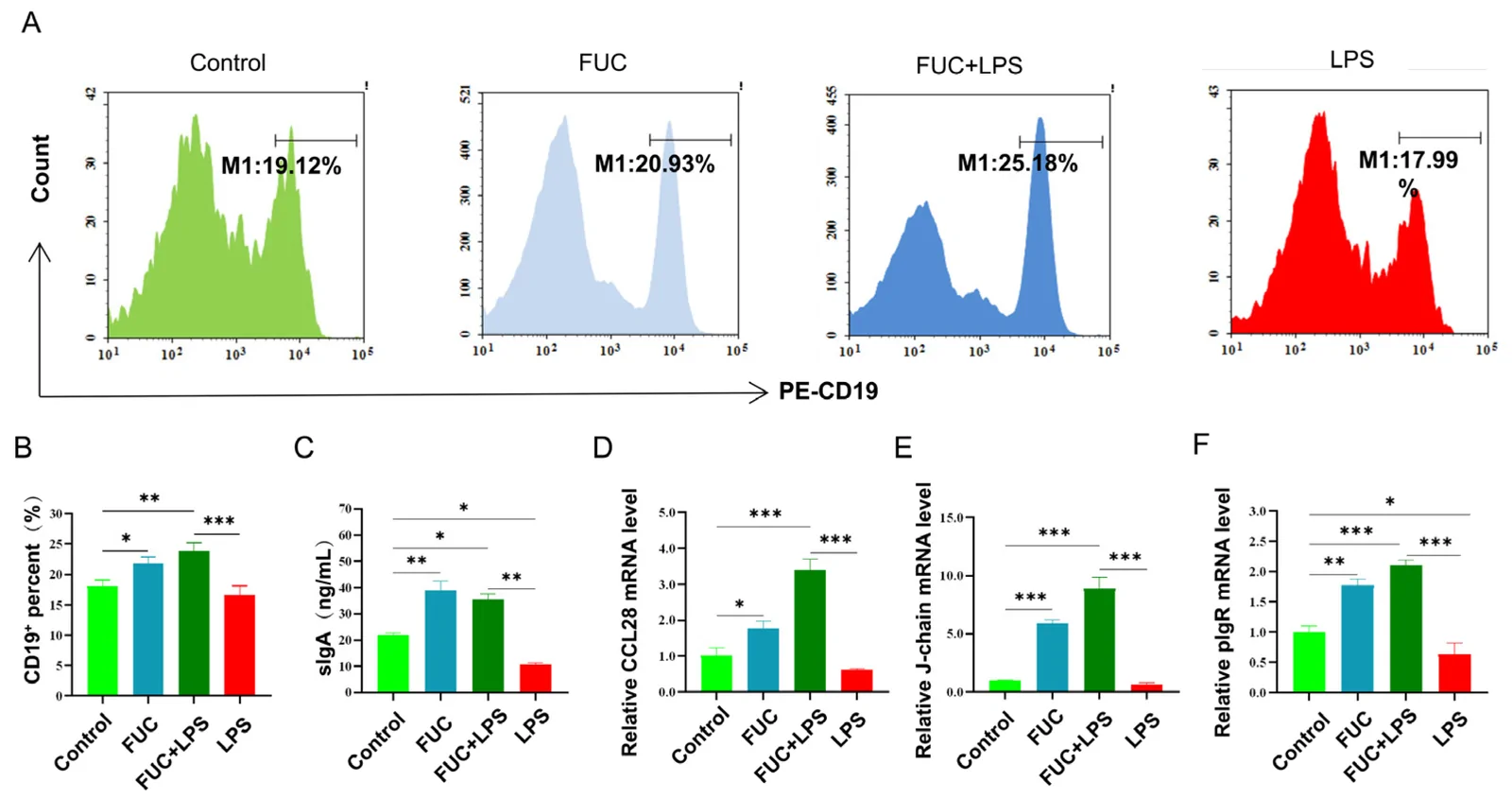
FUC enhances humoral immunity by promoting B cell activation and sIgA production. (**A**) Flow cytometric analysis of B lymphocytes in the intestinal tissue of mice. (**B**) Effects of FUC on B lymphocytes in the small intestine of mice. (**C**) Secretory IgA (sIgA) levels in the intestinal tissue of mice. (**D**–**F**) Expression levels of sIgA-related cytokines in the intestinal tissue of mice. * *p* < 0.05, ** *p* < 0.01, *** *p* < 0.001.

The proportion of B cells in the intestinal lamina propria was measured by flow cytometry. Compared with the control group, FUC treatment alone increased the proportion of intestinal lamina propria B cells by 1.81% (*p* < 0.05; Figure 6A,B). In the LPS-induced model, the proportion of B cells in the FUC + LPS group was 7.19% higher than that in the LPS group (*p* < 0.001).

ELISA results showed that FUC treatment significantly increased intestinal sIgA levels (*p* < 0.05; Figure 6C) and reversed the LPS-induced reduction in sIgA secretion (*p* < 0.01). In addition, FUC significantly increased the mRNA expression of J-chain, pIgR, and CCL28, which are associated with sIgA secretion and transport (*p* < 0.05; Figure 6D–F). In LPS-challenged mice, FUC pretreatment significantly restored the expression of pIgR and other sIgA-related molecules that were reduced by LPS (*p* < 0.001).

Collectively, these findings suggest that FUC may enhance intestinal mucosal humoral immunity by promoting B cell recruitment, regulating sIgA-related factors, and increasing sIgA secretion. These effects may contribute to the attenuation of LPS-induced intestinal inflammation.

## 3. Discussion

Fucoidan is an important class of sulfated bioactive polysaccharides derived from brown algae. It has attracted increasing attention because of its anti-inflammatory, antiviral, antioxidant, antitumor, and immunomodulatory activities. Fucoidan may also act as a prebiotic and contribute to intestinal homeostasis [19]. These properties suggest its potential application in local mucosal immune regulation.

Previous studies have shown that FUC can promote DC maturation and enhance antigen presentation, thereby regulating immune responses [20]. FUC has also been reported to restore macrophage phagocytic activity and T cell activity in immunosuppressed mice. These effects were accompanied by increased IFN-γ and IL-12 expression and reduced IL-4 and IL-10 expression, indicating a shift toward Th1-related immune responses [6]. In addition, FUC can enhance NK cell cytotoxicity by increasing the secretion of perforin and granzyme B, thereby improving antitumor activity [21]. These findings indicate that FUC can regulate both innate and adaptive immune responses. Notably, the biological activity of fucoidan is closely related to its source and structural features.

In the present study, a sulfated polysaccharide with an average molecular weight of approximately 73.5 kDa was extracted and purified from kelp collected from the Lianjiang coast of Fujian, China. Structural characterization identified this polysaccharide as fucoidan. Given that fucoidan composition varies with algal source and extraction conditions, the reproducibility of our findings across different batches requires further validation.

The molecular weight of our fucoidan (73 kDa) is in the medium range compared with previously reported values, such as 7.12 kDa for fucoidan from *Sargassum latifolium* [22] and 61.5/167.6 kDa for two fractions from *Sargassum pallidum* reported by Liu et al. [23]. Medium-molecular-weight fucoidans generally strike a balance between maintaining structural integrity and ensuring receptor accessibility, which is favorable for immune cell signaling modulation and bioactivity expression [24].

The sulfate content of this fucoidan was determined to be 24.16%, which is higher than the levels reported for many previously studied fucoidans. This high sulfation degree may facilitate classical electrostatic interactions with immune cell surface receptors, mucus constituents, and cationic proteins, thereby potentiating its regulatory effects on inflammatory cytokines, immune cell activation, and mucosal barrier integrity [25]. Moreover, the uronic acid content of 6.51% may influence the intestinal fate of this polysaccharide, potentially enhancing its accessibility to microbial enzymatic degradation and subsequent immunomodulatory actions [26]. We further examined its effects on DC function and intestinal mucosal immune responses.

LPS is a major component of the outer membrane of Gram-negative bacteria and is widely used to establish inflammatory disease models. Intraperitoneal LPS injection can induce a strong inflammatory response and increase the release of pro-inflammatory cytokines, including TNF-α and IL-1β [27]. This response is often accompanied by immune organ dysfunction, impaired intestinal tight junctions, and intestinal barrier damage. In severe cases, systemic inflammatory responses may occur.

In this study, an LPS-induced acute intestinal inflammation model was established by intraperitoneal injection of LPS at 3 mg/kg. FUC pretreatment reduced LPS-induced intestinal pathological injury, improved the spleen-to-body weight and intestine-to-body weight ratios, and preserved intestinal tissue structure. At the molecular level, LPS increased the expression of IL-1β, TNF-α, and IL-6, whereas FUC pretreatment reduced the expression of these inflammatory factors. These findings are consistent with previous reports showing that fucoidan attenuates LPS-induced neuronal injury and acute lung injury [28,29]. They suggest that FUC may alleviate LPS-induced tissue injury by limiting excessive inflammatory responses.

Previous studies have proposed several mechanisms through which FUC and other bioactive polysaccharides may reduce intestinal inflammation. These mechanisms include regulation of Toll-like receptor (TLR) signaling, inhibition of NLRP3 inflammasome activation, restoration of epithelial tight junctions and mucus layers, and modulation of the gut microbiota and its metabolites, such as short-chain fatty acids (SCFAs) [6,30,31,32]. Our findings further support the protective role of FUC in intestinal inflammation and barrier injury.

DCs are central regulators of intestinal mucosal immunity. They are distributed in Peyer’s patches and the intestinal lamina propria. Through pattern recognition receptors (PRRs), DCs recognize and capture antigens and regulate intestinal immune responses. MHC-II and CD86 are commonly used markers of DC maturation [33]. After antigen capture, DCs migrate to secondary lymphoid organs. They process antigens and present antigen-derived peptides through MHC-II molecules. CD86 provides a costimulatory signal for naïve T cell activation and the initiation of antigen-specific immune responses [34].

Several natural polysaccharides, including polysaccharides from *Ganoderma lucidum*, *Lentinula edodes*, and *Achyranthes bidentata*, have been reported to increase CD86 and MHC-II expression on DCs and promote DC maturation. In the present study, FUC significantly increased CD86 and MHC-II expression on BMDCs. This result is consistent with a previous report on FUC-induced DC maturation [35]. In addition, FUC increased the proportion of mature DCs in the intestinal lamina propria and Peyer’s patches. This effect was also observed under LPS challenge. These findings suggest that FUC may support DC-mediated mucosal immune regulation under both physiological and inflammatory conditions.

Immature DCs have strong antigen uptake capacity, including dextran uptake, but show limited antigen-presenting activity. During maturation, antigen uptake gradually decreases, whereas antigen processing and presentation are enhanced [36]. In our study, FUC reduced FITC-dextran uptake by DCs in a concentration-dependent manner. This result supports the maturation-promoting effect of FUC. T cell proliferation is also commonly used to evaluate the antigen-presenting capacity of DCs [37]. FUC-treated DCs promoted T cell proliferation, especially at concentrations of 100 and 200 μg/mL. These results indicate that FUC may enhance DC-mediated antigen presentation and T cell activation.

TLR signaling may contribute to FUC-induced DC maturation. Liu et al. [35] reported that the TLR4 inhibitor TAK-242 significantly reduced FUC-induced DC maturation, suggesting the involvement of TLR4. In our in vitro experiments, FUC increased the mRNA expression of TLR4, MyD88, and NF-κB in BMDCs. These findings suggest that TLR4/MyD88/NF-κB signaling may participate in FUC-induced DC maturation.

Previous studies have shown that fucoidans from different brown algae can activate NF-κB signaling through TLR2 or TLR4 in HEK293 cells. Their affinity for TLRs may vary according to structural characteristics [38]. In the present study, no significant change in TLR2 expression was observed. This difference may be related to the structural features of FUC or the cell type used.

The in vivo results suggest that FUC regulates TLR4-related signaling in a context-dependent manner. In healthy mice, FUC increased the expression of TLR4 and NF-κB. Under LPS challenge, FUC significantly reduced the expression of TLR4, MyD88, and NF-κB compared with the LPS group. Thus, FUC did not simply activate or inhibit this pathway; instead, it appeared to modulate the expression of TLR4-related signaling molecules according to the immune environment. Further studies using receptor blockers or gene-specific approaches are needed to confirm the direct role of TLR4 in FUC-mediated DC regulation.

Mature DCs regulate T cell and B cell responses through antigen presentation, costimulatory signals, and cytokine secretion. CD4^+^ T cells coordinate immune responses and support host defense, whereas CD8^+^ T cells directly contribute to the elimination of target cells [39]. In this study, FUC increased the proportion of CD4^+^ T cells in the intestinal lamina propria. FUC pretreatment also further increased the proportions of CD4^+^ and CD8^+^ T cells in LPS-challenged mice. These findings suggest that FUC supports cellular immune responses in the intestinal mucosa.

CD4^+^ T cells can differentiate into several subsets, including Th1, Th2, Th17, and Treg cells. DC-derived cytokines are important regulators of this process. IL-12 promotes Th1-associated responses through T-bet and supports IFN-γ production. Th2-associated responses are regulated by GATA-3 and IL-4. IL-10 is an important anti-inflammatory cytokine that limits excessive inflammation [40]. Under specific inflammatory conditions, IL-6 and TGF-β may promote Th17-associated responses through STAT3 and RORγt. In contrast, in the absence of strong IL-6-mediated inflammatory signals, TGF-β favors Foxp3-associated Treg responses [41,42].

In the LPS-induced inflammatory environment, FUC reduced the excessive expression of IL-1β, TNF-α, and IL-6. FUC also increased T-bet and Foxp3 expression, whereas GATA-3 and RORγt expression showed no significant changes. In addition, FUC pretreatment increased IL-10, IL-12, IFN-γ, and TGF-β expression and reduced IL-17 expression compared with the LPS group. These findings are consistent with enhanced Th1- and Treg-associated immune regulation and restrained Th17-associated inflammatory activity. Therefore, FUC may help restore Th1/Th17/Treg-related immune balance in the intestinal mucosa. However, these results reflect changes in transcription factors and cytokines. Direct measurements of Th1, Th17, and Treg cell frequencies will be needed to further confirm changes in T cell differentiation.

In addition to T cell regulation, mature DCs can influence B cell responses and intestinal sIgA production. In the present study, FUC increased the proportion of B cells in the intestinal lamina propria under physiological conditions. It also increased the B cell proportion in LPS-challenged mice. Previous evidence suggests that DCs can promote IgA responses in B cells [43].

The production and transport of sIgA require several coordinated processes. CCL28 promotes the mucosal recruitment of IgA-secreting plasma cells. J chain participates in the formation of polymeric IgA, and pIgR mediates the transepithelial transport of sIgA [44]. In this study, FUC increased intestinal sIgA levels and upregulated the mRNA expression of CCL28, J chain, and pIgR. These findings suggest that FUC may enhance sIgA production and transport in the intestinal mucosa. Similar effects have been reported for other functional polysaccharides that promote DC maturation and sIgA responses [45].

In the present study, the signaling pathways activated by FUC in dendritic cells were assessed mainly at the mRNA level and require protein-level validation. Furthermore, previous studies have shown that FUC is resistant to digestion in the upper gastrointestinal tract and is fermented by specific gut microbes, leading to altered microbial composition and production of bioactive metabolites [46]. This provides a basis for future research into how FUC modulates intestinal immune cells via the gut microbiota.

Overall, FUC may enhance intestinal mucosal immunity by promoting DC maturation, increasing B cell accumulation, and supporting sIgA-related immune responses. These effects may contribute to the attenuation of LPS-induced intestinal immune dysregulation.

## 4. Materials and Methods

### 4.1. Experimental Animals

Female C57BL/6 mice (6–8 weeks old) were purchased from SPF Biotechnology Co., Ltd. (Suzhou, Jiangsu, China; animal license No. SYXK (Su) 2022-0012). All animal experiments were approved by the Animal Ethics Committee of Fujian Agriculture and Forestry University (Approval No. FZCASFAFU21015) and were conducted in accordance with the Regulations for the Administration of Affairs Concerning Experimental Animals of China.

### 4.2. Materials and Reagents

*Saccharina japonica* samples were collected from the coastal area of Lianjiang, Fujian Province, China. The samples were washed, dried, and ground into powder before use.

Recombinant mouse GM-CSF (rmGM-CSF) and IL-4 (rmIL-4) were obtained from PeproTech (Rocky Hill, NJ, USA). RNA reverse transcription reagents were purchased from Promega (Madison, WI, USA), and PerfectStar Green SuperMix was obtained from TransGen Biotech (Beijing, China).

Fluorescein isothiocyanate (FITC)-conjugated anti-mouse CD11c, FITC-CD103 (F1090C), PE-MHC-II (F0990D), APC-CD86 (F0994E-50T), anti-mouse CD16/32 monoclonal antibody, FITC-CD3 (F1013C-50T), PerCP-CD4 (E-AB-F1097F), APC-CD8 (AN00576E), PE-CD19 (E-AB-F0986D), and staining buffer were purchased from Elabscience Biotechnology Co., Ltd. (Wuhan, China).

FITC-dextran and type IV collagenase were obtained from Sigma-Aldrich (St. Louis, MO, USA). Percoll cell separation solution, DNase I, and dithiothreitol (DTT) were purchased from Beijing Solarbio Science & Technology Co., Ltd. (Beijing, China). The sIgA ELISA kit was obtained from Jiangsu Meimian Industrial Co., Ltd. (Yancheng, Jiangsu, China). Absorbance values were determined using a SpectraMax190 absorbance microplate reader (Molecular Devices, San Jose, CA, USA). All fucoidan preparations employed in this study originated from the same batch.

### 4.3. Extraction and Purification of Fucoidan

Fucoidan was extracted and purified from kelp with minor modifications based on a previously reported method [47]. Briefly, kelp powder was defatted using 95% ethanol at a solid-to-liquid ratio of 1:20 (g/mL, *w*/*v*). After removal of the precipitate, the residue was extracted with distilled water at a solid-to-liquid ratio of 1:50 (g/mL, *w*/*v*) at 90 °C for 5 h (Figure 1A). The extract was collected, concentrated by rotary evaporation, and treated with CaCl_2_ to remove alginate by precipitation. Three volumes of absolute ethanol were added to the supernatant, and the mixture was incubated overnight at 4 °C to precipitate crude polysaccharides. The precipitate was dissolved in sterile water and deproteinized using the Sevag method. After dialysis (molecular weight cutoff: 10 kDa) to remove small molecules, the solution was filtered through a 0.22 μm membrane. The crude polysaccharide solution (5 mL, 250 mg) was loaded onto a DEAE-Sepharose Fast Flow column (Ruida Henghui, Beijing, China). After washing with water, stepwise elution was performed with 0, 0.1 M, 0.4 M, 0.8 M and 1.2 M NaCl at 1 mL/min. Fractions of 5 mL were collected and monitored by the phenol-sulfuric acid method. The major fraction was pooled, dialyzed (MWCO: 3500 Da), and lyophilized. The total sugar content of each fraction was determined using the phenol–sulfuric acid method [48], and the elution profile was generated. The target fraction was collected, dialyzed for desalination, and lyophilized to obtain purified fucoidan.

### 4.4. Physicochemical Characterization of Fucoidan

The total sugar content of FUC was determined using the phenol–sulfuric acid method. Briefly, FUC solution was mixed with phenol solution and concentrated sulfuric acid, followed by incubation at room temperature in the dark. After color development, absorbance was measured at 490 nm, and glucose was used as the standard for quantification.

The fucose content was determined using the cysteine–sulfuric acid method [49]. Briefly, the FUC sample was initially subjected to hydrolysis with distilled water. Subsequently, the hydrolysate was reacted with cysteine-hydrochloride-sulfuric acid reagent. Following color development in a water bath and subsequent cooling, the absorbance was recorded at 396 nm and 430 nm, with L-fucose employed as the reference standard for calibration.

The sulfate content was determined using the barium chloride–gelatin method [50]. FUC solution was reacted with barium chloride to form barium sulfate precipitates. After mixing and incubation, absorbance was measured at 360 nm, and sulfate content was calculated based on a standard curve.

The uronic acid content was measured using the m-hydroxydiphenyl method [51,52]. Samples were reacted with sulfuric acid in a boiling water bath, followed by the addition of m-hydroxydiphenyl reagent, and the mixture was then cooled to room temperature. Absorbance was measured at 525 nm using spectrophotometer, and galacturonic acid was used as the standard.

### 4.5. Determination of Molecular Weight

The apparent molecular weight of FUC was determined by gel permeation chromatography (GPC). Briefly, 3–5 mg of FUC sample was dissolved in 1 mL ultrapure water and allowed to stand for 1 h. The solution was filtered through a 0.22 μm membrane before analysis. The analysis was performed using a Waters E2695 GPC system (Waters, Milford, MA, USA) equipped with a refractive index detector and a PL aquagel-OH MIXED-M column. A 0.1 M NaNO_3_ solution was used as the mobile phase at a flow rate of 1.0 mL/min. The column temperature was maintained at 40 °C. The apparent molecular weight distribution was calculated using a calibration curve generated with polyethylene glycol (PEG) standards of known molecular weights.

### 4.6. Monosaccharide Composition Analysis

The monosaccharide composition of FUC was determined by high-performance liquid chromatography (HPLC). FUC samples were hydrolyzed with 2 M trifluoroacetic acid (TFA) at 110 °C and subsequently derivatized with 1-phenyl-3-methyl-5-pyrazolone (PMP). The derivatives were analyzed using an Nertsil ODS-SP C18 column (250 mm × 4.6 mm, 5 μm, GL Sciences Inc., Tokyo, Japan). The mobile phase consisted of 0.1 M ammonium acetate solution (pH 5.5) and acetonitrile (83:17, *v*/*v*). The flow rate was 1 mL/min, the column temperature was maintained at 30 °C, and the detection wavelength was set at 250 nm. Monosaccharides were identified by comparison with standard compounds, including mannose (Man), rhamnose (Rha), glucose (Glc), galactose (Gal), xylose (Xyl), and fucose (Fuc). Quantification was performed based on peak areas.

### 4.7. Fourier-Transform Infrared Spectroscopy (FT-IR)

Lyophilized FUC samples were thoroughly mixed with potassium bromide (KBr), ground into a fine powder, and pressed into pellets. FT-IR spectra were recorded using a Fourier-transform infrared spectrometer (Thermo Scientific Nicolet iN10 MX, Waltham, MA, USA) over the range of 4000–400 cm^−1^. The characteristic functional groups of FUC were identified based on the obtained spectra.

### 4.8. In Vitro Culture and Treatment of Mouse Bone Marrow-Derived Dendritic Cells

Bone marrow-derived dendritic cells (BMDCs) were generated from the femurs and tibias of female C57BL/6 mice (6–8 weeks old). Bone marrow cells were flushed with PBS and cultured in RPMI 1640 complete medium supplemented with 10% fetal bovine serum (FBS), 1% penicillin–streptomycin, 20 ng/mL GM-CSF, and 20 ng/mL IL-4.

Cells were seeded at a density of 1 × 10^6^ cells per dish and cultured at 37 °C in a humidified atmosphere containing 5% CO_2_. Fresh medium containing the same concentrations of cytokines was replaced every two days. On day 6, differentiated BMDCs were collected for subsequent experiments. To evaluate the effects of FUC on BMDCs, cells were resuspended at 1 × 10^6^ cells/mL and seeded into 6-well plates. Cells were divided into five groups: control group, LPS stimulation group, and FUC-treated groups. The control group received an equal volume of medium, the LPS group was treated with 50 ng/mL LPS, and FUC-treated groups were supplemented with FUC at final concentrations of 50, 100, and 200 μg/mL [24,53]. Cells were cultured for 48 h at 37 °C with 5% CO_2_ and collected for further analysis.

### 4.9. Detection of DC Phagocytic Activity

The antigen uptake capacity of DCs was evaluated using a FITC-dextran phagocytosis assay. Briefly, treated cells were collected and resuspended in pre-warmed complete medium containing 1 mg/mL FITC-dextran. Cells were incubated at 37 °C in a humidified atmosphere with 5% CO_2_ for 1 h in the dark. After incubation, the reaction was immediately terminated by adding pre-chilled PBS, and cells were washed thoroughly to remove extracellular FITC-dextran. Cells were then stained with PE-conjugated anti-CD11c antibody. The fluorescence intensity of FITC in CD11c^+^ cells was analyzed by flow cytometry. The phagocytic capacity of DCs was evaluated based on the percentage of FITC-dextran-positive CD11c^+^ cells.

### 4.10. Mixed Lymphocyte Reaction

A mixed lymphocyte reaction (MLR) assay was performed to assess the capacity of FUC-treated DCs to promote splenic T cell proliferation. Briefly, DCs subjected to different treatments were incubated with 25 μg/mL mitomycin C at 37 °C for 30 min to inhibit DC proliferation and were then washed thoroughly. Splenic T cells were isolated from female C57BL/6J mice (6–8 weeks old). Mitomycin C-treated DCs were co-cultured with splenic T cells at a DC ratio of 1:5 in 96-well plates for 72 h. T cell proliferation was assessed using the Cell Counting Kit-8 (CCK-8) assay (Beyotime Biotechnology, Shanghai, China).

### 4.11. Animal Experiments

Forty C57BL/6 mice were randomly divided into four groups (*n* = 10 per group): the control group (CON), FUC-treated group (FUC), LPS-induced model group (LPS), and FUC intervention plus LPS-treated group (FUC + LPS). Mice in the FUC and FUC + LPS groups were orally administered FUC at a dose of 200 mg/kg daily, while mice in the CON and LPS groups received an equal volume of saline. All treatments were continued for 14 consecutive days.

On day 13, mice in the LPS and FUC + LPS groups were intraperitoneally injected with LPS (3 mg/kg) to establish an acute endotoxemia-induced intestinal inflammation model. Mice in the CON and FUC groups received an equal volume of saline.

Twenty-four hours after LPS administration, body weight was recorded, and mice were euthanized by ether inhalation. Intestinal tissues were collected. Ileum, jejunum, and colon tissues were immediately fixed in 4% paraformaldehyde for histological analysis, while remaining samples were rapidly frozen and stored at −80 °C for further analysis.

### 4.12. Isolation of Intestinal Mucosal Immune Cells

After euthanasia, the entire small intestine was rapidly collected. Mesenteric tissues and attached adipose tissues were carefully removed. Peyer’s patches and the remaining intestinal tissues were separately placed in pre-chilled Hank’s balanced salt solution (HBSS without Ca^2+^/Mg^2+^).

Based on a previously described method [54] with minor modifications, tissues were minced and digested in HBSS containing type IV collagenase (1 mg/mL) and DNase I (0.1 mg/mL) at 37 °C for 30–45 min with gentle shaking to obtain single-cell suspensions. The digested cell suspension was filtered through a 70 μm cell strainer. Intestinal lymphocytes were enriched by density gradient centrifugation using 40% and 80% Percoll solutions. The isolated cells were used for subsequent flow cytometric analysis.

### 4.13. Flow Cytometry Analysis

The expression of DC surface markers was analyzed by flow cytometry. Briefly, cells were first incubated with anti-mouse CD16/32 monoclonal antibody at 4 °C for 15 min in the dark to block nonspecific Fc receptor binding. Cells were subsequently stained with fluorochrome-conjugated antibodies, including FITC-anti-CD11c, PE-anti-MHC-II, and APC-anti-CD86, at 4 °C for 30 min in the dark. After staining, cells were washed twice with PBS containing 2% FBS. The maturation status of DCs was evaluated by analyzing the mean fluorescence intensity (MFI) of MHC-II and CD86 in CD11c^+^ cells.

### 4.14. Quantitative Real-Time PCR (qRT-PCR) Analysis

Total RNA was extracted from tissues or cells using the TransZol Up Plus RNA Kit (TransGen Biotech Co., Ltd., Beijing, China). RNA (TransGen Biotech, Beijing, China) concentration and purity were determined before reverse transcription. First-strand cDNA was synthesized from 1 μg of total RNA using a reverse transcription kit according to the manufacturer’s instructions. Quantitative PCR was performed using PerfectStar Green qPCR SuperMix on a QuantStudio real-time PCR system. The amplification program was as follows: initial denaturation at 95 °C for 30 s, followed by 40 cycles of 95 °C for 5 s and 60 °C for 30 s. β-actin was used as the internal reference gene, and relative gene expression levels were calculated using the 2^−ΔΔCt^ method. Primer sequences are listed in Supplementary Table S1.

### 4.15. ELISA Analysis

Approximately 10 cm of small intestine tissue was longitudinally opened, and intestinal mucosa was gently scraped using a glass slide into 1 mL PBS. The tissue samples were homogenized and centrifuged at 10,000× *g* for 15 min at 4 °C. The supernatants were collected for analysis [55]. The concentration of secretory IgA (sIgA) was determined using a commercial ELISA kit (Baolai Biotechnology, Yancheng, Jiangsu, China) according to the manufacturer’s instructions.

### 4.16. Statistical Analysis

Flow cytometry data were analyzed using NovoExpress software (version 1.6.1). Other experimental data were statistically analyzed using GraphPad Prism 9. All data are presented as mean ± standard deviation (SD). Statistical differences among multiple groups were analyzed using one-way analysis of variance (ANOVA), followed by Tukey’s multiple comparison test. Differences were considered statistically significant at *p* < 0.05.

## 5. Conclusions

A sulfated polysaccharide with a weight-average molecular weight of approximately 73.5 kDa was extracted and purified from kelp. It contained fucose as the predominant monosaccharide and was rich in sulfate groups and uronic acid. These structural features may be related to its biological activity.

The in vitro and in vivo results showed that FUC promoted DC maturation and enhanced antigen-presenting and immunoregulatory functions. These effects were associated with TLR4/MyD88-related signaling. In the LPS-induced acute intestinal inflammation model, FUC reduced the expression of pro-inflammatory cytokines, including TNF-α and IL-1β, and improved intestinal mucosal barrier function. FUC also increased CD4^+^ and CD8^+^ T cell responses and regulated Th1/Th17/Treg-related immune balance. In addition, FUC increased B cell accumulation and sIgA secretion.

Taken together, the results of this study indicate that FUC exerts a protective effect against intestinal inflammation by synergistically modulating mucosal barrier integrity, cellular immune responses, and humoral immunity. This mechanistic insight lays the foundation for future studies to elucidate the regulatory networks of FUC in intestinal mucosal immunity and provides a critical theoretical basis for its development as a novel immunomodulator and delivery vehicle.

## Data Availability

The data presented in this study are available on request from the corresponding author.

## Supplementary Materials

The following supporting information can be downloaded at: https://www.mdpi.com/article/10.3390/md24080274/s1, Table S1: Primer sequences used for qRT-PCR analysis.
