# Supplementary material for: Saccharina japonica-Derived Fucoidan Protects Intestinal Immune Homeostasis by Modulating Dendritic Cell Function and Alleviating LPS-Induced Acute Enteritis
Source: Mar Drugs. 2026 Aug 6;24(8):274. doi: 10.3390/md24080274 (PMC13514979; doi:10.3390/md24080274)
Supplement: Supplementary file 1 [file marinedrugs-24-00274-s001.zip › marinedrugs-4447650-supplementary.pdf]

Table S1. Primer sequences used for qRT-PCR analysis.

Primer name	Primer sequence (5′–3′)
**IL-12**	F: AAGACATCACACGGGACCAAAC<br>R: CACAGGGTCATCATCAAAGACGT
**IL-4**	F: CTTCCAAGGTGCTTCGCATA<br>R: GATGAATCCAGGCATCGAAA
**IL-6**	F: TAGTCCTTCCTACCCCAATTTCC<br>R: TTGGTCCTTAGCCACTCCTTC
**TGF-β**	F: CTGCTGACCCCCACTGATAC<br>R: GGGGCTGATCCCGTTGATT
**Claudin-1**	F: AGATACAGTGCAAAGTCTTCGA<br>R: CAGGATGCCAATTACCATCAAG
**Occludin**	F: TTGGCTACGGAGGTGGCTATGG<br>R: CCTTTGGCTGCTCTTGGGTCTG
**ZO-1**	F: AACCCGAAACTGATGCTGTGGATAG<br>R: CGCCCTTGGAATGTATGTGGAGAG
**TNF-α**	F: CAGGCGGTGCCTATGTCTC<br>R: CGATCACCCCGAAGTTCAGTAG
**IL-1β**	F: GGGCCTCAAAGGAAAGAATC<br>R: TACCAGTTGGGGAACTCTGC
**IFN-γ**	F: TCAGCAACAACATAAGCGTCAT<br>R: GACCTCAAACTTGGCAATACTCA
**IL-23**	F: CTCTCGGAATCTCTGCATGC<br>R: ACCATCTTCACACTGGATACG
**IL-17**	F: GCGCAAAAGTGAGCTCCAGA<br>R: ACAGAGGGATATCTATCAGGG

**IL-10**	F: GCTGGACAACATACTGCTAACCGACTC<br>R: CCTTGATTTCTGGGCCATGCTTCTC
**TLR4**	F: ATGGCATGGCTTACACCACC<br>R: GAGGCCAATTTTGTCTCCACA
**MyD88**	F: CTGGCCTTGTTAGACCGTGA<br>R: TGGGACACTGCTTTCCACTC
**NF-κB**	F: TCCGGGAGCCTCTAGTGAGAA<br>R: TCCATTTGTGACCAACTGAACG
**T-bet**	F: CAACAACCCCTTTGCCAAAG<br>R: TCCCCCAAGCAGTTGACAGT
**FoxP3**	F： CCCAGGAAAGACAGCAACCTT<br>R: TTCTCACAACCAGGCCACTTG
**GATA-3**	F: CGGGTTCGGATGTAAGTCGAGG<br>R: GATGTCCCTGCTCTCCTTGCTG
**RORγt**	F: CCGCTGAGAGGGCTTCAC<br>R: TGCAGGAGTAGGCCACATTACA
**CCL-28**	F: GTGTGTGGCTTTTCAAACCTCA<br>R: TGCATGAACTCACTCTTTCCAG
**pIgR**	F: ATGAGGCTCTACTTGTTCACGC<br>R: CGCCTTCTATACTACTCACCTCC
**J-chain**	F: TGACGACGAAGCGACCATTC<br>R: TTCAAAGGGACAACAATTCGGA
**β-actin**	F: ACTGTGCCCATCTACG<br>R: ATGTCACGCACGATTT
